# Supplementary material for: Erg4 Is Involved in Ergosterol Biosynthesis, Conidiation and Stress Response in Penicillium expansum
Source: J Fungi (Basel). 2023 May 13;9(5):568. doi: 10.3390/jof9050568 (PMC10220616; doi:10.3390/jof9050568)
Supplement: Supplementary file 1 [file jof-09-00568-s001.zip › jof-2355832-supplementary.pdf]

Supplementary Materials

Supplementary Figure S1. Construction of *erg4*s gene deletion mutants.

Supplementary Figure S2. PCR results of gene knockout mutants of *erg4s* in *P. expansum*. The amplification fragments of *erg4A*-up, *erg4B*-up, *erg4C*-up, *erg4A*-down, *erg4B*-down, *erg4C*-down from *P. expansum*, respectively (A). The PCR identification of Δ*erg4A* (B), Δ*erg4B* (C) and Δ*erg4C* (D).

Supplementary Table S1. The primers used for the construction and identification of gene deletion mutants and complementation strains.

Supplementary Table S2. Primers used for RT-qPCR.

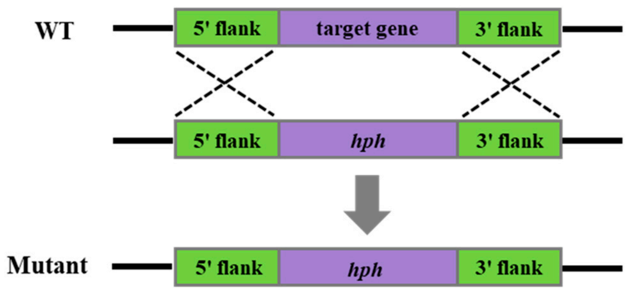
WT
5' flank
target gene
3' flank
5' flank
hph
3' flank
Mutant
5' flank
hph
3' flank

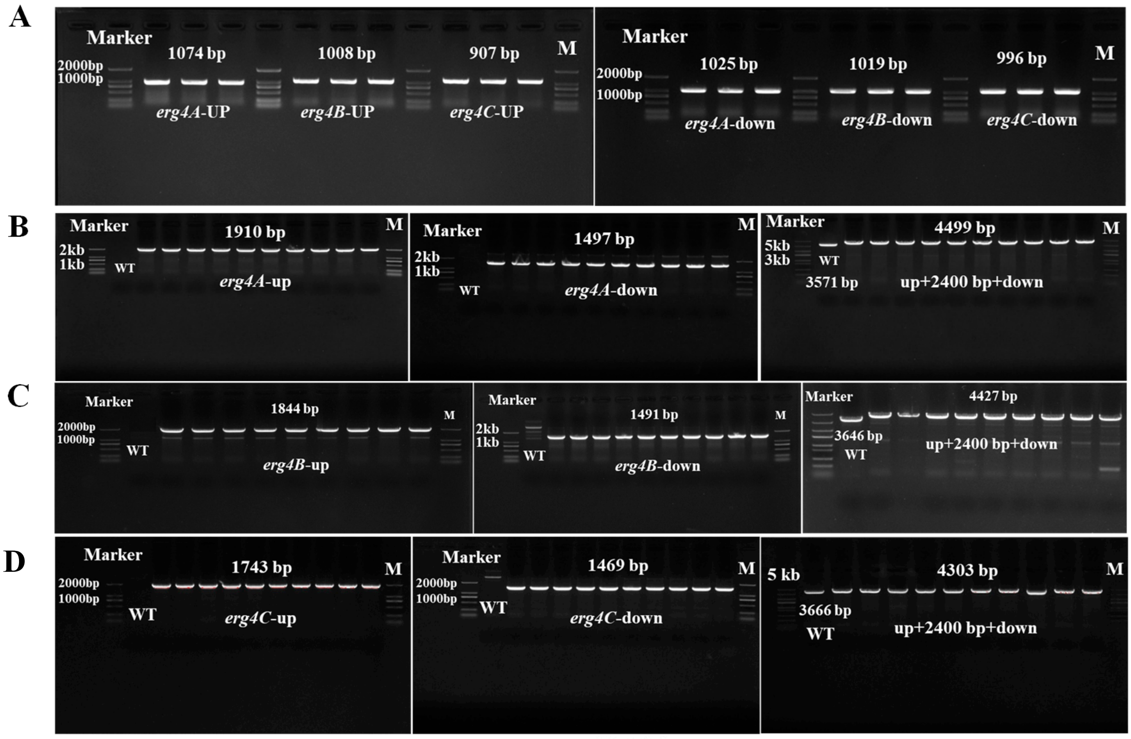
A
Marker
2000bp
1000bp
1074 bp
1008 bp
907 bp
M
erg4A-UP
erg4B-UP
erg4C-UP
Marker
2000bp
1000bp
1025 bp
1019 bp
996 bp
M
erg4A-down
erg4B-down
erg4C-down
B
Marker
2kb
1kb
WT
1910 bp
erg4A-up
M
Marker
2kb
1kb
WT
1497 bp
erg4A-down
M
Marker
5kb
3kb
WT
4499 bp
3571 bp
up+2400 bp+down
M
C
Marker
2000bp
1000bp
WT
1844 bp
erg4B-up
M
Marker
2kb
1kb
WT
1491 bp
erg4B-down
M
Marker
4427 bp
3646 bp
WT
up+2400 bp+down
D
Marker
2000bp
1000bp
WT
1743 bp
erg4C-up
M
Marker
2000bp
1000bp
WT
1469 bp
erg4C-down
M
5 kb
4303 bp
3666 bp
WT
up+2400 bp+down
M

Name	Primer sequence (5' - 3')
erg4A-up-F	ACAGCTATGACCATGATTACGAATTCAGTAAACTCCTCCAACGAA
erg4A-up-R	GATCCCCGGGTACCGAGCTCGAATTCCTCACTCCTGGCAGTATGT
erg4A -down-F	TTGCCTAACTCGGCGCGCCGAAGCTT GTTCAAGTTTGTTTCGTCG
erg4A -down-R	GTAAAACGACGGCCAGTGCCAAGCTTGAGGGCACCAATAATAAGA
erg4B-up-F	ACAGCTATGACCATGATTACGAATTC CGTCACGGTCACAAAGGA
erg4B-up-R	GATCCCCGGGTACCGAGCTC GAATTC CACTGGCTGCACGGATAT
erg4B-down-F	TTGCCTAACTCGGCGCGCCGAAGCTT AGGAGAAGTGCGAGAAGA
erg4B-down-R	GTAAAACGACGGCCAGTGCCAAGCTT GACTAACCCGTCACCATT
erg4C-up-F	ACAGCTATGACCATGATTACGAATTC TTACCTTGCCAATCTTCC
erg4C-up-R	GATCCCCGGGTACCGAGCTC GAATTC GACCACCATTCAGCCATA
erg4C-down-F	TTGCCTAACTCGGCGCGCCGAAGCTT CTCAATGAAGCCGGGACG
erg4C-down-R	GTAAAACGACGGCCAGTGCCAAGCTT AGCCTCGATGTTTGTTCG
TrpC-F	TAGAGTAGATGCCGACCGG
OliC-R	CTGAAAGCACGAGATTCTTC
erg4A-F	TCAACTCCATCACATCACAAGAGCTCATGGAGGACCAAGTCAAATC
erg4A-R	AGCTCCTCGCCCTTGCTCACTCTAGAAAAAAGAGAAATTGCAGTAG
erg4B-F	TCAACTCCATCACATCACAAGAGCTCATGCCCTCCAAAAAGGACTC
erg4B-R	AGCTCCTCGCCCTTGCTCACTCTAGAGTAAATTCCAGGAATGATGC
erg4C-F	GCATGGACGAGCTGTACAAGGAGCTCATGGATCGTCCCGGCTTCAT
erg4C-R	ATGGAGCTATTAAATCACTATCTAGATTAGAAGACATACTATAGGA
CX1	GGAGA CGTAT TTAGGTGCTA
CX2	TGAACTTCA GGGTCAGCTT
erg4A-GFP-F	ATGGAGGACCAAGTCAAATC
erg4A-GFP-R	AAAAAGAGAA ATTGCAGTAG
erg4B-GFP-F	ATGCCCTCCAAAAAGGACTC
erg4B-GFP-R	GTAAATTCCA GGAATGATGC
erg4C-GFP-F	ATGGATCGTC CCGGCTTCAT
erg4C-GFP-R	TTAGAAGACA TAAGGAATGA

Name	Primer sequence (5' - 3')
erg4A-F	TCCACGAATGTTCGGAATCC
erg4A-R	AGCTCAAACCCAAGAGCATGA
erg4B-F	CGGCGCTTTATGATACCAATG
erg4B-R	GGGAAGGAAGACCTGCAAAAA
erg4C-F	GCATTCCCATCAATCAAAGCA
erg4C-R	CCGGGAAGAAGCACGTAACA
β-tubulin-F	CTCCAGCTCGAGCGTATGAAC
β-tubulin-R	GGCTCCAAATCGACGAGAAC
